# Understanding the effect of producers’ attitudes, perceived norms, and perceived behavioral control on intentions to use antimicrobials prudently on New York dairy farms

**DOI:** 10.1371/journal.pone.0222442

**Published:** 2019-09-11

**Authors:** Amy K. Vasquez, Carla Foditsch, Stéphie-Anne C. Dulièpre, Julie D. Siler, David R. Just, Lorin D. Warnick, Daryl V. Nydam, Jaap Sok

**Affiliations:** 1 Department of Population Medicine and Diagnostic Sciences, College of Veterinary Medicine, Cornell University, Ithaca, New York, United States of America; 2 Dyson School of Applied Economics and Management, Cornell University, Ithaca, New York, United States of America; 3 Department of Social Sciences, Business Economics, Wageningen University, Wageningen, The Netherlands; Ross University School of Veterinary Medicine, SAINT KITTS AND NEVIS

## Abstract

Understanding farmers’ behavior, motivations, and perceptions toward antimicrobial use can influence how veterinarians translate research into practice and guide effective ways of implementing protocols. A multidisciplinary team investigated behavioral tendencies of New York dairy farmers toward antimicrobial use by administering a survey modeled with the reasoned action approach. This approach is a framework from social psychology containing the constructs attitude, perceived norms, and perceived behavioral control, and is used in combination with structural equation modeling to determine what drives intentions. Multiple indicators and multiple causes (MIMIC) models were then used to determine the effects of beliefs on their underlying constructs. The objective of the study was to provide direct and indirect measures of the constructs using survey data to determine importance of and associations with intention to use antimicrobials prudently. The structural equation model indicated that perceived behavioral control explained intention. Thus, farmers who feel capable of prudent use expressed positive intentions. Attitude and perception of others also had influence to a lesser extent. MIMIC models showed that the most important attributes of instrumental attitude were increasing profitability, decreasing risk of residues, and increasing herd health. Contributing attributes of affective attitude were job satisfaction, decreasing resistance, and increasing milk production. For perceived norms, the attributes were opinions/approval of family and peers, veterinarians, and milk processors. Finally, for perceived behavioral control, attributes focused on saving money on labor and treatment, ability to fit into the daily routine, and effectiveness with veterinary guidance. In conclusion, the best approach for adoption of practices might be presentation of examples of successful strategies by other producers, particularly in peer groups. In addition, veterinarians should provide the tools and guidance needed to produce economic gain, reduction of risks associated with residues and resistance, and positive experiences when using the tactics.

## Introduction

Antimicrobial resistance is a public health concern worldwide [1]. Populations of bacteria might naturally become resistant; however, the overuse and misuse of antimicrobials by human medical and animal production systems has accelerated the evolution of resistance [2–5]. In the agricultural sector, non-prudent use of antimicrobials as therapeutics or prophylactics can have a negative impact on sustainability by reducing drug efficacy, as pathogenic microorganisms can develop resistance to antimicrobials, leading to cycles of prolonged use of the same drug or supplemented use of alternative antimicrobials in order to find an effective treatment [6]. One system that has been scrutinized is the conventional US dairy farm. A standardized estimate of exposure to antimicrobial drugs on dairy farms in Wisconsin showed that dairy cows received 5.43 defined daily doses per adult cow per year, with 80% attributed to mastitis treatment and prevention [7].

Policies have been established, for example, in the Netherlands, that forbid preventative use of antimicrobials on dairy farms for purposes such as prophylactic treatment of all cows prior to their non-milking dry period [8]. In the US, an acknowledged step in limiting antimicrobial resistance is to ensure correct usage of these drugs when needed [9]. Strategies to use antimicrobials prudently, such as selective treatment of mastitis based on pathogen characteristics have been explored and many studies have shown no appreciable negative outcomes [10–13]. Similar studies have been done for selective treatment of cows at dry-off, with the dry period being a high-risk period for new infections [14–18]. Despite favorable results in these studies, promotion of these practices at conferences attended by veterinarians, and publication of extension articles in industry related magazines, a survey by the US National Animal Health Monitoring System in 2014 indicated that 87.3% of cows with clinical mastitis are treated with antimicrobials and over 90% of cows are treated at dry-off with a long-acting antimicrobial [19]. Additionally, a recent survey on dairies in Washington State indicated that a large proportion of participants had positive intentions and behaviors toward reducing antimicrobial use: these participants also 1) understood that antibiotics that worked well in the past are becoming less effective; 2) selected the correct definition of resistance [20]. No social psychology modeling studies have been published in the US to evaluate what factors contribute to these intentions and eventual actions. Understanding dairy farmers’ behavior, motivations, and perceptions toward antimicrobial use in the US, where policies and practices differ from those implemented in Europe, can influence how veterinarians, industry professionals, and extension experts translate research into practice and guide effective ways of implementing protocols.

In this study, a multidisciplinary team investigated behavioral tendencies of New York dairy farmers toward antimicrobial use by administering a survey modeled with the reasoned action approach. This approach is a framework from social psychology containing the constructs attitude toward the expected outcome of the behavior (*A*), perceived norms or beliefs about what others expect them to do in relation to the behavior (*PN*), and perceived behavioral control (perception of the ease or difficulty of a behavior; *PBC*), and is used in combination with structural equation modeling (SEM) to determine what drives intentions (*I*). These frameworks have been explored previously in agriculture to understand intentions to vaccinate, diversify production, reduce antimicrobial usage, or to engage in sustainable practices [21–24]. Targeted communication and education concerning the judicious use of antimicrobials is extremely important to decrease the emergence of resistant bacteria, and thus can contribute to sustainable agriculture. Results of the explored analysis can facilitate approaches or interventions that promote prudent use of antimicrobials on dairy farms.

## Materials and methods

### Survey methods and population

#### Questionnaire distribution

This manuscript included a questionnaire and survey of human subjects. This research was submitted to, and qualified for exemption under, the Institutional Review Board at Cornell University (IRB # 1711007612). All participants provided a signed informed consent. With the aim of reaching the largest number of New York dairy farms possible, and to avoid bias of retrieving data only from farms affiliated with Cornell University, we collaborated with the New York State Department of Agriculture and Markets. A printed copy of the survey was mailed in February 2018 to all 4,970 dairy farms listed with the department. A copy of the survey is provided as supporting information (S1 Appendix). Initial pages obtained demographic information and the actual questionnaire had four pages introduced by definitions of ‘antibiotic resistance’ and ‘prudent use’. The last page collected names and emails for compensation for participation. All data collected was anonymous and confidential; survey responses and email addresses were not linked. To preserve the confidentiality of survey respondents, authors have not publicly shared the data.

#### The reasoned action approach

The reasoned action approach is an integrative framework that contains the subcomponents attitude, perceived normative beliefs, and perceived behavioral control, and is often used in social behavior analysis to determine what drives people’s intentions and behaviors [25]. Statistical models can be represented by *B* (given behavior), *I* (intention to perform the behavior), *A* (attitude), *PN* (perceived norms), and *PBC* (perceived behavioral control). Attitudes are often based on core values and perception of good/bad behaviors and their associated positive or negative outcomes. Perceived norms are social pressures to perform a behavior derived from the expectations of contacts, the public, industry partners, etc. Perceived behavioral control is the belief in self-efficacy or confidence to complete a behavior. The theory can be represented by:

$$B\sim I=f_{I}(A,PN,PBC)$$ (1)

An expanded model consisting of two *A* components, instrumental attitude (*A*_*I*_) and affective attitude (*A*_*A*_), two *PN* components, injunctive norm (*N*_*I*_; the perceptions of what specific referents think the person should do) and descriptive norm (*N*_*D*_; the perceived behavior of others), and finally, two *PBC* components (*PBC*_*A*_ *PBC*_*C*_; autonomy and capacity) has been proposed [26], such that:

$$PN=f_{PN}(N_{I},N_{D})$$ (2)

$$A=f_{A}(A_{I},A_{A})$$ (3)

$$PBC=f_{PBC}(PBC_{A},PBC_{C})$$ (4)

A short description is well stated by Lam et al.: ‘if someone is actually willing to solve an issue, if he is positively influenced by important peers and if he has the feeling he can control and perform his actions, he will have a positive intention and probably will change his behavior’ [27]. Thus determination of the importance of each component in a model for intention to use antimicrobials prudently will direct private and public entities to approach behavioral change by placing more emphasis on changing attitudes, promoting peer involvement or competition, or by encouraging or providing resources that promote the confidence to do so.

### Methods of analysis

The constructs can be measured directly and indirectly. Direct measurements are more strongly associated with intentions and model estimates in regards to associations can indicate the relative importance between each construct in predicting a behavior [28]. In this study, SEM is used to generate model estimates using direct measurements, and answers the question, is it primarily attitude, norms, or perceived behavioral control that drives the behavior? Indirect measurements assess the underlying beliefs behind the direct measures that explain intention. These beliefs can be identified and analyzed to determine what drives intentions of producers to participate in practices of prudent antimicrobial use. Multiple indicators multiple cause (MIMIC) modeling is used in this study to identify these important determinants from indirect measurements. It answers the questions, what or who influences attitude, normative beliefs, and perceived control so interventions can target these entities.

#### Direct measurements of reasoned action approach constructs and SEM

Within the survey, actions were defined in terms of target, action, context, and time using the “TACT” principles [29]. For example, each question was preceded by ‘the following questions refer to decisions *[action]* made on your farm *[context]* over the next two years *[time]*.’ The statement that followed mentioned the *target* (prudent antibiotic use). For the constructs, three statements were used to directly measure producers’ intentions (*I*) to use antimicrobials prudently, and another twelve statements to directly measure the constructs *A* (5), *PN* (3), and *PBC* (4), as shown in Table 1. The constructs were measured using a five-point bipolar Likert-like scale (scores of 1 to 5), with gradations of negative to positive response choices.

**Table 1:** Survey statements for direct measurements representing each construct of a reasoned action approach for prudent use of antimicrobials on dairy farms.

Construct	Variable	Description of the statement	
Attitude	*a*_1_	Indicate whether using antibiotics prudently would be …	…disadvantageous-advantageous^a^
	*a*_2_		…unsatisfying-satisfying^b^
	*a*_3_		…unnecessary-necessary^a^
	*a*_4_		…unimportant-important^a^
	*a*_5_		…unpleasant-pleasant^b^
Perceived norms	*pn*_1_	Most people who have something to do with my farm expect me to use antibiotics prudently	
	*pn*_2_	The people in the dairy industry whose opinions I value would approve of me using antibiotics prudently	
	*pn*_3_	Most people who are important to me think that I should use antibiotics prudently	
Perceived behavioral control	*pbc*_1_	I have the possibility to use antibiotics prudently^c^	
	*pbc*_2_	If I wanted to, I could use antibiotics prudently^d^	
	*pbc*_3_	It is up to me whether I use antibiotics prudently^d^	
	*pbc*_4_	I am confident that I can use antibiotics prudently^c^	
Intention	*i*_1_	I will try to use antibiotics prudently	
	*i*_2_	I intend to use antibiotics prudently	
	*i*_3_	I plan to use antibiotics prudently	

In the original survey, all questions were preceded with ‘The following questions refer to decisions made on your farm over the next two years’.

^a^Statements reflect affective/experiential attitude.

^b^Statements reflect instrumental/economic attitude.

^c^Statements reflect perceived self-capacity/self-efficacy.

^d^Statement reflects self-autonomy.

Using values from each respondent, SEM was used to determine correlational and causal relationships among the constructs. The use of “causal” as a description of these relationships does not attribute any significance to the term “cause” other than the fact that the indicators *determine* the latent variable [30, 31]. SEM is similar to regression analysis but offers the ability of inclusion of more and latent variables, several pathways modeled simultaneously, correlation of errors, and analysis of direct and indirect effects. The goal within a final model was to define the construct using multiple variables represented by survey answers and also correct for measurement errors. This is done using a two-step approach [32], recognizing that direct measurement statements were inherently assigned to constructs based on the reasoned action approach model/theory. The first step was to use confirmatory factor analysis to estimate a measurement model that would determine which variables were appropriate and representative of each construct. Correlation was allowed between all constructs. The second step was to use the subsequent inputs for attitude, perceived norms, and perceived behavioral control as exogenous variables in a structural model to test their relationships on the endogenous variable *I*. Several model specifications were run to assess multicollinearity; R-squared values were used to explain variance. All statistical modeling was performed in STATA v. 15 (StataCorp LLC, College Station, TX), using a significance level of *P* < 0.05 for inclusion of variables in the model. The impact of constructs is presented as the regression coefficients (β-parameters) in each model.

Overall model fit was assessed using the goodness-of-fit measures most commonly applied in published SEM analyses, along with their acceptable cut-off values: the root mean square error of approximation (RMSEA) ≤ 0.06, Bentler’s comparative fit index (CFI) ≥ 0.95 and the standardized root mean square residual (SRMR) ≤ 0.08 [33–35]. Construct and discriminant validity of the hypothesised latent constructs was assessed with the average variance extracted (AVE) and composite reliability (CR) statistics [32, 36].

#### Indirect measurements and MIMIC models

Within the same survey, indirect questions were asked to determine the attributes that contribute to each individual construct. The components of the indirect questions were identified using peer-reviewed publications on agricultural surveys that identified attitude indicators related to milk production, economics, job satisfaction, and herd health [6, 24, 37] as well as previous literature on antimicrobial use on dairies identifying indicators such as risk of residues and resistance [37]. There were two main questions for each construct containing several sub-statements. The resulting scores for the related sub-statements of each question would result in a multiplicative composite: the product of a belief statement with the outcome evaluation statement. This method of measurement is based on the expectancy-value theory, which assumes that there are expectations as well as values or beliefs that affect subsequent behavior [38, 39]. For the attitude construct, one question pertained to strength of the attitudinal belief about the attribute in the statement. Each statement began with ‘please indicate how likely using antibiotics prudently will…’ and each indicator (*e*.*g*., ‘increase milk production’) followed. Responses were then selected on a 5-point scale of ‘not at all likely’ to ‘very likely.’ The subsequent question pertained to the evaluation of the attribute in the statement. Here each statement began with, ‘how important are the following motives for using antibiotics prudently…’ and each indicator followed (*e*.*g*., ‘increased milk production of your cows’). Responses were then selected on a 5-point scale of ‘not at all important’ to ‘extremely important.’

For perceived norms, one question pertained to the producers’ perception of a list of particular referents identified in previous literature as being important contributors to decisions made on farms. These referents included family members/friends, neighbors, veterinarians, their milk plant, consumers, scientists/researchers, and government regulators [6, 24, 27, 37, 40]. The question asked ‘do you think the following people would approve or disapprove of you using antibiotics prudently’ and respondents indicated ‘strong disapproval’ to ‘strong approval’ on a 5 point scale. Additionally, this question offered the ability to select ‘I don’t know.’ The second question of the series determined the participant’s identification with the referent and asked ‘how important is the referent’s opinion to you in regards to prudent use of antibiotics?’ Answers indicated level of importance from ‘not important at all’ to ‘extremely important.’

Finally, for perceived behavioral control, the following factors were identified: fitting into a daily routine, saving money on treatment and labor, feasibility as knowledge of the task was known, ease of seeing animal health changes occur in the short time, ease of seeing changes in revenue in the short term, effectiveness with veterinary guidance, and compensation with premiums [6, 24]. The first in the series of questions inquired about the likeliness of the control factor when the behavior is practiced. For example one statement was, ‘please indicate how likely using antibiotics prudently will fit into your daily work routine’. Respondents selected likeliness on a 5-point scale. A second question inquired about the power of each control factor in the perceived personal capability to perform prudent use practices. For example a statement read, ‘will the following points make it easier or more difficult for you to use antibiotics prudently…’ with the control statement (*e*.*g*. ‘fitting into your daily work routine’) following. Respondents selected answers on a 5-point scale from ‘extremely difficult’ to ‘extremely easy’.

The first question in each series was scored 1 through 5, while the second question in each series was scored -2 to 2. Indirect measures from each of the three psychological constructs were used to build three models for analyzing the main recurring beliefs following previous guidelines [38, 39]. In each model, a multiplicative composite was created for each attribute (the values for each of the two indirect statements pertaining to each attribute were multiplied), with a possible score for each attribute ranging from -10 to 10. These multiplicative composites were then analyzed as a set of causal indicators explaining each latent variable in multiple regression models called MIMIC models (multiple indicator and multiple causes) [41]. MIMIC models were recently used and are formally described in a similar approach for determining farmer’s beliefs in voluntary vaccination schemes for Bluetongue in Dutch dairy cattle [42]. The resulting models in this study will describe the major determinants for attitudes surrounding prudent use, the influential relationships or referents that play a role, and the drivers of autonomy or capacity associated with intentions to use antimicrobials prudently.

For both SEM and MIMIC modeling, surveys with missing data were not included. The number of ‘don’t know’ ticks in the perceived norms construct was tabulated and assessed. Additionally, within all MIMIC models, variance inflation factors were assessed, as values greater than 5 indicate a level of multicollinearity suggestive of redundant variables [43]. This was non-problematic in the current analysis.

All statistical modeling for indirect measures was also performed in STATA; nonsignificant indicators that exceeded a Type I error level of 10% were removed using backwards step-wise removal. Model fit was assessed using χ^2^ statistics, RMSEA, CFI, and SRMR [36]. The impact of causal indicators is presented as the regression coefficients (γ-parameters) in each model.

## Results

### Respondent demographics and farm characteristics

More than 500 envelopes were non-deliverable as addressed, leaving 4,417 surveys delivered in February 2018. Most surveys were returned within one month; the final response was recorded in May 2018. One hundred-ten surveys were returned by respondents who indicated that their facility was no longer a functioning dairy (*e*.*g*. retired farms) or the facility was inappropriately placed on the state list (*e*.*g*. heifer raisers, non-dairy farm). We retrieved 411 surveys back from functioning dairy facilities; assuming the remainder of the surveys were correctly addressed, this corresponds to a response rate of 9.5% (411/4307). The majority of people responded on paper (382), and only 29 completed the survey online. Of the 411, 364 were conventional dairy farms (88.6%) and 47 were organic (11.4%). Only conventional farms were considered in the analysis. Five additional questionnaires were excluded from the analysis due to a majority of missing data. The overall effective sample size was 359 (8.3%).

Demographics of respondents, farm characteristics, management practices, veterinary involvement, and antimicrobial use practices can be seen in the S1 Table. Over 90% of respondents were farm owners, and approximately 30% were greater than 60 years old. Approximately 60% of farms had regular routine veterinary involvement, but frequency of visits ranged from every other month or less (15.3% of farms) to twice or more per month (22.9% of farms). In regards to antimicrobial use practices, only 1/3 of respondents treated every case of clinical mastitis with antimicrobials and 2/3 of dairy respondents treated all cows with antimicrobials at dry-off.

### Descriptive statistics and modeling of direct measures

Skewness and kurtosis fell within the boundaries of Kline [44]. Response values for direct measurements of attitude towards prudent use were high, indicating positive attitudes toward prudent use; all means were greater than 3.8 on a 5 point scale (3.8–4.5; Table 2). Individual correlations, which were all less than 0.54 suggested that *a*_2_ and *a*_5_, questions reflective of instrumental considerations (‘satisfaction and pleasantness’), differed from the affective or experiential considerations in the remaining statements (‘advantageous’, ‘necessary’, ‘important’) (Tables 1 and 2).

**Table 2:** Sample correlation matrix with means and standard deviations of the variables representing the constructs attitude (*a*_1_-*a*_5_), perceived norms (*pn*_1_-*pn*_3_), perceived behavioral control (*pbc*_1_-*pbc*_4_), and intention (*i*_1_-*i*_3_).

**Variable**		**1**	**2**	**3**	**4**	**5**	**6**	**7**	**8**	**9**	**10**	**11**	**12**	**13**	**14**	**15**
**1**	***a***_**1**_	1														
**2**	***a***_**2**_	**0.53**^1^	1													
**3**	***a***_**3**_	**0.72**	**0.52**	1												
**4**	***a***_**4**_	**0.66**	**0.48**	**0.81**	1											
**5**	***a***_**5**_	**0.48**	**0.65**	**0.49**	**0.51**	1										
**6**	***pn***_**1**_	0.37	0.27	0.40	0.38	0.28	1									
**7**	***pn***_**2**_	0.35	0.25	0.36	0.36	0.29	**0.71**	1								
**8**	***pn***_**3**_	0.37	0.3	0.38	0.42	0.32	**0.71**	**0.72**	1							
**9**	***pbc***_**1**_	0.39	0.33	0.44	0.38	0.28	0.39	0.34	0.36	1						
**10**	***pbc***_**2**_	0.27	0.30	0.34	0.31	0.28	0.32	0.26	0.29	**0.70**	1					
**11**	***pbc***_**3**_	0.13	0.16	0.25	0.28	0.22	0.12	0.17	0.17	**0.35**	**0.42**	1				
**12**	***pbc***_**4**_	0.38	0.31	0.43	0.38	0.27	0.34	0.29	0.37	**0.64**	**0.54**	**0.46**	1			
**13**	***i***_**1**_	0.32	0.22	0.32	0.28	0.31	0.36	0.34	0.37	0.65	0.63	0.41	0.57	1		
**14**	***i***_**2**_	0.46	0.30	0.45	0.38	0.36	0.47	0.40	0.42	0.65	0.47	0.33	0.65	**0.79**	1	
**15**	***i***_**3**_	0.45	0.26	0.44	0.38	0.32	0.45	0.35	0.42	0.60	0.50	0.30	0.70	**0.78**	**0.86**	1
**Mean**^2^		4.5	4.1	4.4	4.5	3.8	4.3	4.5	4.3	4.5	4.4	4.5	4.5	4.4	4.5	4.5
**Std. Dev.**		0.9	1.0	0.8	0.8	1.1	0.9	0.8	0.9	0.8	0.9	0.9	0.8	1.0	0.9	0.9

Attitude, perceived norms, perceived behavioral control, and intention are constructs in the reasoned action approach, an integrative framework for behavior analysis. Each variable listed is representative of a question in a behavioral survey querying current practices and future intentions for prudent use of antimicrobials on NY dairy farms (n = 359).

^1^Bold and shaded values indicate intra-construct correlation.

^2^The range of individual respondent values for each variable was 1 to 5.

Unfortunately, the formulated survey only queried for response values for injunctive normative beliefs and did not include questions for both injunctive and descriptive normative beliefs. Response values for direct measurements of normative beliefs were greater than 4 (4.3–4.5), indicating strong agreement that individuals in the industry or of importance to the respondent influence their thoughts regarding prudent use. Here individual correlations for all questions were > 0.70.

Regarding perceived behavioral control responses, mean rank scores were each greater than 4 (4.4–4.5), indicating that farmers did perceive that they had the ability to use antimicrobials prudently. Individual correlations within this construct were low (majority < 0.60). This was further addressed in the final measurement model (see below).

All variables except for those related to perceived behavioral control were only weakly correlated with intention, with 2/3 of the correlations between the two constructs greater than 0.50. Finally, intentions toward prudent use were positive, as mean values for all response questions were approximately 4.5.

Confirmatory analysis resulted in removal and respecification of variables. Fit indices indicated a poor fit for the saturated model; modification indices and standardized residuals suggested problems with covariance in components of attitude. Theoretically, this can be explained by the idea that these statements made up the specific dimension affective attitude introduced earlier. Analysis confirmed that an alternative model using two constructs was advantageous, concluding that *a*_1_, *a*_3_, and *a*_4_ made up the instrumental attitude construct (*A*_*I*_), while *a*_2_ and *a*_5_ made up the affective attitude construct (*A*_*A*_) construct.

Evaluation of the measurement model also indicated that the high correlation between the perceived behavioral control and intention constructs was not ideal. A solution resulted after removal of *pbc*_1_, *pbc*_4_, and *i*_1_. Likely, the two *pbc* statements were reflective of the capacity or self-efficacy dimension of perceived behavioral control *(PBC*_*C*_*)*, while the remaining two statements ‘if I wanted to’ and ‘it is up to me’ reflected perceived autonomy (*PBC*_*A*_), or beliefs that a person has control over the behavior. Changes in the construct resulted in less ideal values for AVE and CR, but modification indices and standardized residuals indicated no issues with any of the final perceived behavioral control components.

Though initial fit components for the intention construct were high, the inclusion of *i*_1_ indicated high modification indices and covariance with measures of perceived behavioral control as well as high standardized covariance residuals with *i*_3_ (9.7). Subsequent removal of *i*_1_ produced more acceptable AVE and CR. Theoretically, the statement ‘I will try to’ indicated a different level of commitment of intention, as compared to the other statements ‘I intend to’ and ‘I plan to’. The fit indices of the final and saturated models in addition to the CR and AVE values for each of the construct modifications can be seen in the S2 Table.

**Fig 1:**
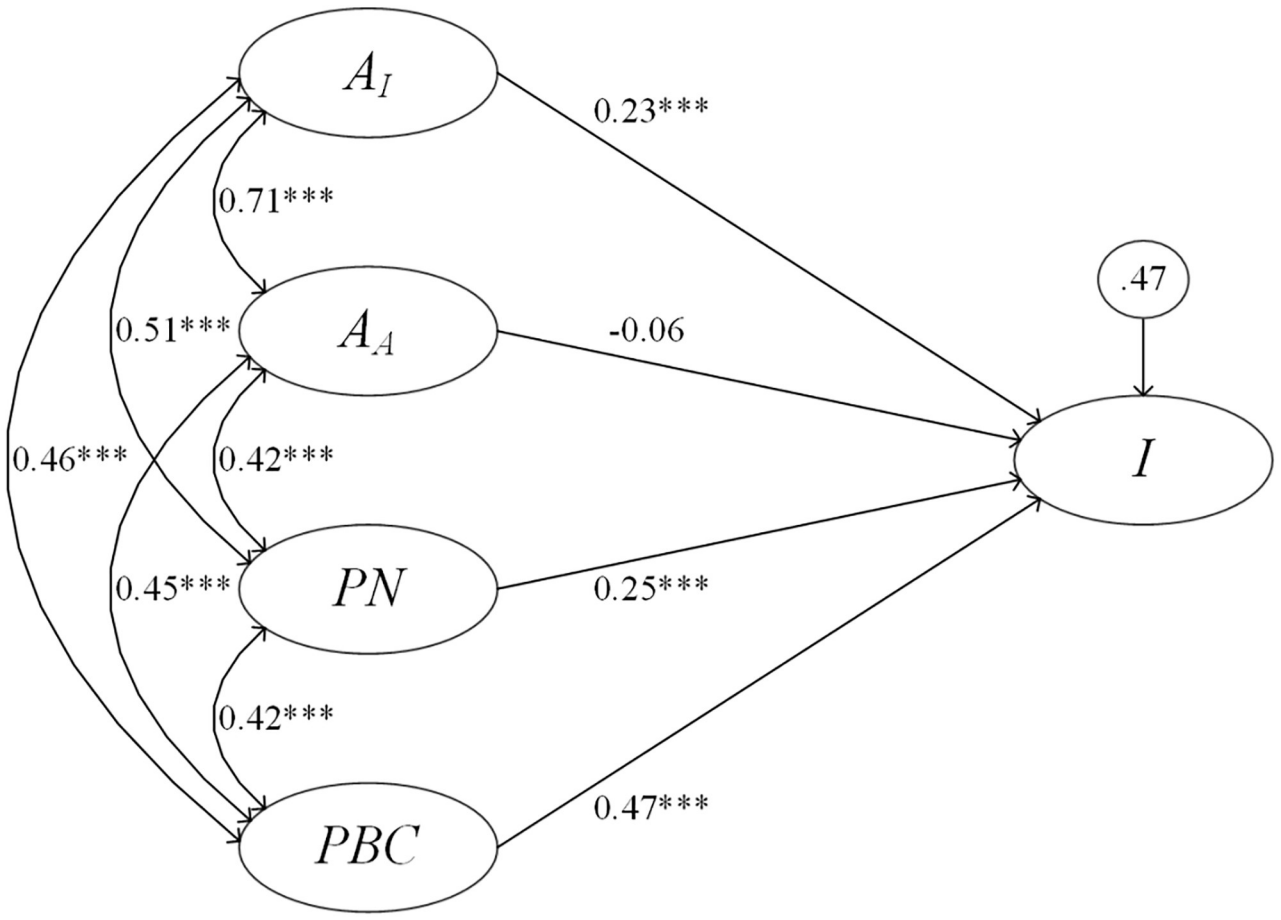
Diagram of the structural equation model of future intentions for prudent use of antimicrobials on NY dairy farms. Instrumental attitude (*A*_*I*_), affective attitude (*A*_*A*_), perceived norms (*PN*), perceived behavioral control (*PBC*), and intention (*I*) are constructs in the reasoned action approach, an integrative framework for behavior analysis. Each construct is represented by questions in a behavioral survey administered to NY dairy farms (n = 359). Ellipses represent constructs, circles represent error, straight arrows represent direct relationships with respective regression coefficients, and curved arrows represent correlation. *** *P* < 0.001.

Fig 1 shows a standardized path diagram for the causal relationships between the exogenous constructs *A*_*I*_, *A*_*A*_, *PN*, and *PBC* on the endogenous construct *I*. The strongest relationship was of *PBC* on *I*, suggesting that the main determinant of intention to use antimicrobials prudently is perceived behavioral control. Discrepancies in direction of relationships (negative and positive) as well as statistical significance of each regression relationship (*β*) in the model suggested the presence of multicollinearity among the constructs. The sources of collinearity in the final model were explored by building several model specifications (Table 3).

**Table 3:** Structural model specifications of selected constructs.

		*Βeta*-estimates^1^ of:				
		1	2	3	4	
Model	Includes	*Instrumental Attitude*	*Affective Attitude*	*Perceived Norms*	*Perceived Behavioral Control*	R^2^
**A**	**1**	0.53*				0.28
**B**	**2**		0.42*			0.17
**C**	**1, 2**	0.47*	0.09			0.29
**D**	**3**			0.54*		0.30
**E**	**1,2,3**	0.31*	0.05	0.36*		0.38
**F**	**4**				0.69*	0.48
**G**	**3, 4**			0.30*	0.53*	0.52
**H**	**1, 2, 3, 4**	0.23*	-0.06	0.25*	0.47*	0.53

Attitude, perceived norms, and perceived behavioral control are constructs in the reasoned action approach, an integrative framework for behavior analysis. Values (direct measurements of constructs) were generated using data from a survey querying current practices and future intentions for prudent use of antimicrobials on NY dairy farms (n = 359).

^1^*Beta*-estimates and *P*-values were generated using structural equation modeling.

R^2^ reflects the variance explained.

*Construct estimates for a model within each row have *P* < 0.05.

Correlation between multiple predictors is apparent, which can be seen as the differences in *β*-estimates for each construct between model specifications when single predictors versus multiple predictors are inserted into the regression. In regards to interpretation of causal relationships on intention, all constructs were important determinants, with separately estimated constructs ranked highest to lowest: perceived behavioral control, perceived norms, instrumental attitude, and affective attitude.

### Descriptive statistics for indirect measures

The proposed indicators as well as the number of responses, correlation, mean values, and standard deviations for each determinant of intention are presented in Tables 4–6, with each attribute corresponding to attitude represented as *att*_*1*_*-att*_*8*_, perceived norms represented as *in*_*1*_*-in*_*7*_, and perceived behavioral control represented as *pbc*_*1*_*-pbc*_*6*_. As all statements were worded in a positive manner and the expected sign for all correlations was positive.

**Table 4:** Descriptive statistics of the causal indicators for attitude, with the belief statement inquiry asking about the likeliness of each indicator and how important each of the motives are for using antibiotics prudently.

Causal indicators	Variable^1^	No.	*Corr*_*AI*_^2^	*Corr*_*AA*_^2^	Mean (SD^3^)
Increased milk production of the herd	*att*_*1*_	347	0.24	0.31	2.6 (4.3)
Cost-effectiveness	*att*_*2*_	347	0.33	0.32	4.6 (4.3)
Job satisfaction	*att*_*3*_	342	0.25	0.50	2.8 (4.3)
Health of the herd	*att*_*4*_	340	0.43	0.30	5.9 (3.8)
Reputation in the dairy industry	*att*_*5*_	343	0.24	0.33	2.8 (4.6)
Increased profitability	*att*_*6*_	346	0.38	0.31	5.5 (3.8)
Decreased risk of residues	*att*_*7*_	342	0.36	0.38	5.3 (4.6)
Decreased risk of antibiotic resistance	*att*_*8*_	344	0.33	0.41	4.9 (4.6)

Attitude is one construct in the reasoned action approach, an integrative framework for behavior analysis. This table describes indirect measures of this construct. Values were generated using data from a survey querying current practices and future intentions for prudent use of antimicrobials on NY dairy farms.

^1^Variables were multiplicative composites of two question types (for example, a belief statement with its associated outcome evaluation statement). The range of individual respondent values for each indicator was -10 to +10

^**2**^Correlation of each composite with the average of the direct measures representing the latent variables instrumental attitude (*A*_*I*_*)* or affective attitude (*A*_*I*_*)*

^3^Standard deviation of the mean

**Table 5:** Descriptive statistics of the perceived norm^1^ referents for approval/importance of opinion in regards to using antibiotics prudently.

Referent	Variable^2^	No.	No. excluding DK^3^	% DK	*Corr*^4^	Mean (SD^5^)
Family members and/or friends	*in*_*1*_	347	330	4.9%	0.48	4.9 (4.0)
Neighboring farmers	*in*_*2*_	347	298	14.1%	0.37	3.2 (3.7)
Veterinarians	*in*_*3*_	346	338	2.3%	0.44	6.4 (3.9)
Milk plant	*in*_*4*_	338	321	5.0%	0.40	5.9 (5.0)
Milk consumers	*in*_*5*_	340	317	6.8%	0.32	4.7 (5.4)
Scientists/researchers	*in*_*6*_	344	279	18.9%	0.35	3.6 (4.0)
Government regulators	*in*_*7*_	343	273	20.4%	0.27	3.4 (4.4)

Perceived norm is one construct in the reasoned action approach, an integrative framework for behavior analysis. This table describes indirect measures of this construct. Values were generated using data from a survey querying current practices and future intentions for prudent use of antimicrobials on NY dairy farms.

^1^Perceived norm was only represented by injunctive norm in the original survey

^2^Variables were multiplicative composites of two question types (for example, a belief statement with its associated outcome evaluation statement). The range of individual respondent values for each indicator was -10 to +10.

^3^DK = ‘Don’t know’ answer choice available to survey respondents.

^4^Correlation of each composite with the average of the direct measures representing the latent variable perceived norm.

^5^Standard deviation of the mean.

**Table 6:** Descriptive statistics for the control beliefs indicating how likely using antibiotics prudently will, or how the following factors will create ease of using antibiotics prudently.

Control factors	Variable^1^	No.	*Corr*^2^	Mean (SD^3^)
Fitting into the daily work routine	*pbc*_*1*_	334	0.35	4.7 (4.2)
Saving money on treatment and labor	*pbc*_*2*_	336	0.37	4.8 (4.1)
Feasibility/knowledge of what needs to be improved	*pbc*_*3*_	329	0.30	3.9 (4.0)
Easy to see/achieve animal health benefits	*pbc*_*4*_	332	0.19	3.7 (4.0)
Easy to see/achieve economic benefits	*pbc*_*5*_	332	0.24	3.2 (3.7)
Effectiveness/availability with veterinary/consultant guidance	*pbc*_*6*_	330	0.21	3.2 (3.7)
Be compensated with premiums	*pbc*_*7*_	325	0.08	1.6 (3.6)

Perceived behavioral control is one construct in the reasoned action approach, an integrative framework for behavior analysis. This table describes indirect measures of this construct. Values were generated using data from a survey querying current practices and future intentions for prudent use of antimicrobials on NY dairy farms.

^1^Variables were multiplicative composites of two question types (for example, a belief statement with its associated outcome evaluation statement). The range of individual respondent values for each indicator was -10 to +10.

^**2**^Correlation of each composite with the average of the direct measures representing the latent variable perceived behavioral control.

^3^Standard deviation of the mean.

The attitudinal belief attribute with the highest mean score was ‘health of the herd’ (5.9), with ‘increased profitability’ and ‘decreased risk of residues’ also having values > 5.0 (Table 4). Eventual assignment into the MIMIC models described these three attributes representative of instrumental (economic) attitude rather than affective (experiential) attitude. Not surprisingly, correlations corresponded with assignment to subcomponents of attitude, with the highest correlation indicators for affective attitude (‘job satisfaction’ and ‘decreased risk of antibiotic resistance’) assigned to affective attitude and the highest correlation indicators for instrumental attitude (‘health of the herd’, ‘increased profitability’, and ‘decreased risk of residues’) assigned to instrumental attitude.

As only the direct measures for injunctive norm were used to represent perceived norms in the SEM models, only the indirect statements regarding injunctive norms were assessed and subsequently analyzed in a MIMIC model. There were seven referents that were explored (Table 5). Only two referents (‘scientists/researchers’ and ‘government regulators’) had greater than 15% ‘don’t know’ ticks. The highest score (6.4) was for ‘veterinarians’ which also had the lowest percent of ‘don’t know’ ticks. The remainder of the referents, in order of decreasing means were ‘milk plant’, ‘family members and/or friends’, ‘milk consumers’, ‘scientists/researchers’, ‘government regulators’, and ‘neighboring farmers.’

For perceived behavioral control, seven control factors were explored (Table 6). The highest mean (4.8) was ‘saving money on treatment and labor’. Surprisingly, another factor that suggested economic benefits, ‘be compensated with premiums’, received the lowest mean (1.6). One explanation might be that farmers do not anticipate that others (*e*.*g*. processors) will provide premiums. The remaining control factors in order of second highest to second lowest were ‘fitting into the daily work routine’, ‘feasibility/knowledge of what needs to be improved’, ‘easy to see/achieve animal health benefits’, and ‘effectiveness/availability with veterinary/consultant guidance’.

### MIMIC models for indirect measures

**Fig 2:**
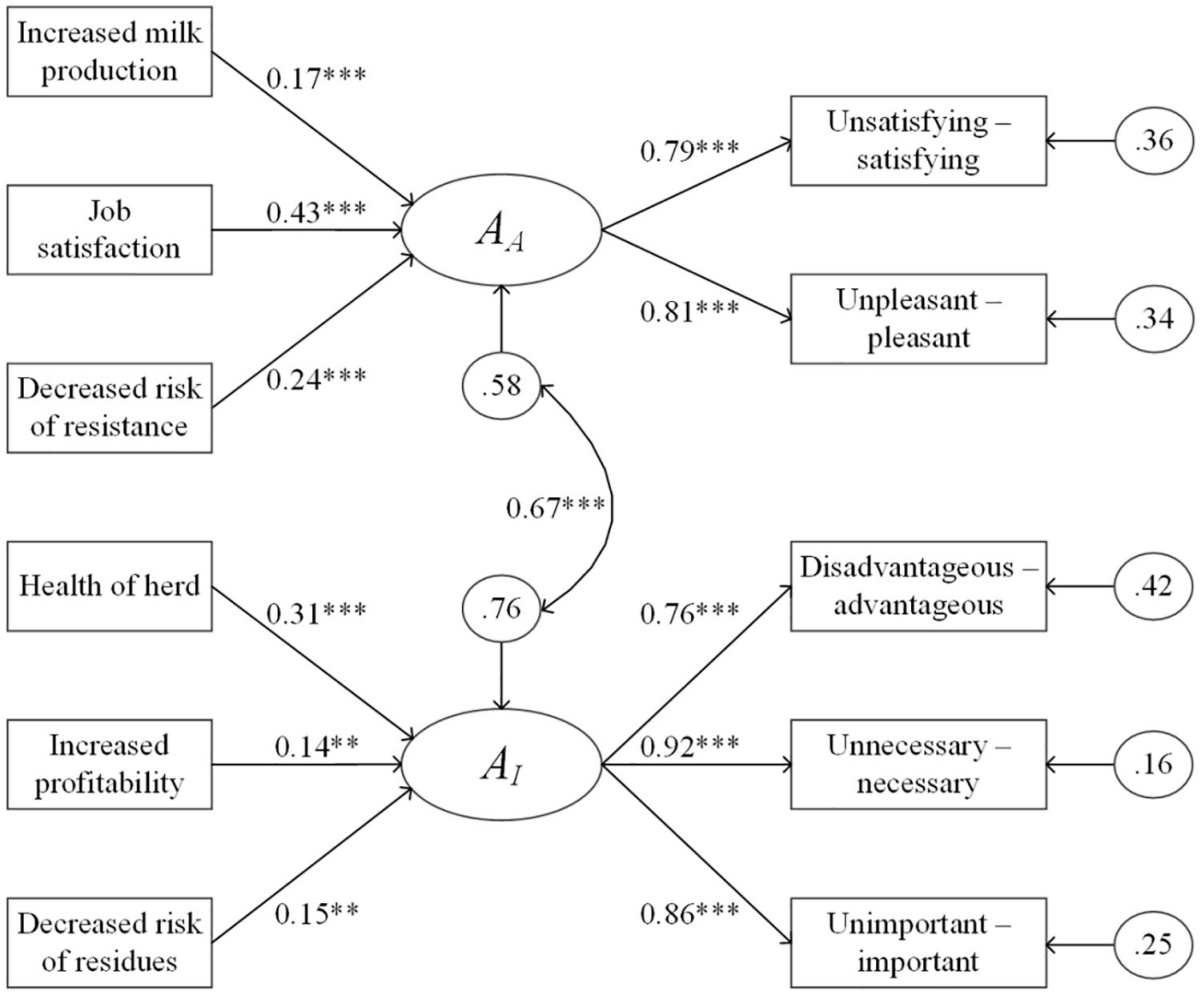
Diagram of the multiple indicators multiple causes model (MIMIC) for attributes of attitude. Instrumental attitude (*A*_*I*_) and affective attitude (*A*_*A*_) are constructs in the reasoned action approach, an integrative framework for behavior analysis. In a MIMIC model, regression coefficients show the impact of the causal relationship of each attribute (*e*.*g*. increased milk production) on the construct, accounting for the relationships to the direct measures (*e*.*g*. unsatisfying-satisfying). Measurements were retrieved as answers to questions in a behavioral survey administered to NY dairy farms. Ellipses represent constructs, circles represent error, straight arrows represent direct relationships with respective regression coefficients, and curved arrows represent correlation. *** *P* < 0.001 ** *P* < 0.05.

Fig 2 shows an example of a path diagram representing the MIMIC model for the indicators causing the attitude construct. The model included all but ‘cost-effectiveness’ as statistically important attributes. Attributes for affective attitude explained 42% of the variance for the construct while attributes for instrumental attitude explained 24%. The attribute for affective attitude with the highest γ-parameter value was ‘job satisfaction’ followed by ‘decreased risk of resistance’ and ‘increased milk production’. The attribute for instrumental attitude with the highest γ-parameter value was ‘health of the herd’ followed by ‘decreased risk of residues’ and ‘increased profitability’, which were nearly equivalent.

Table 7 presents the estimated regression coefficients as well as model fit indices for each of the MIMIC models. The model for referents causing perceived norms included, in order of γ-parameter values, ‘family members/friends’, ‘milk plant’, and ‘veterinarian.’ The fit was not ideal (RMSEA = 0.09) likely due to very few degrees of freedom (6); models with low degrees of freedom always report relatively high RMSEA [45]. The components explained 33% of the variation in the construct. The model for perceived behavioral control had a good fit. Twenty-six percent of the variance was explained by the attributes ‘saving money on treatment/labor’, ‘fitting into the daily routine’, and ‘accessibility with veterinarian/consultant guidance.’

**Table 7:** Estimates of the final multiple indicator multiple cause (MIMIC) models.

Cause	Effect of γ on …				*P*-value
	η_AA_	η_AI_	η_PN_	η_PBC_	
γ_att1_	0.17				0.001
γ_att3_	0.43				<0.0001
γ_att8_	0.24				<0.0001
γ_att4_		0.31			<0.0001
γ_att6_		0.14			0.02
γ_att7_		0.15			0.006
γ_in1_			0.33		<0.0001
γ_in3_			0.16		0.02
γ_in4_			0.20		<0.0001
γ_pbc1_				0.21	0.01
γ_pbc2_				0.30	<0.0001
γ_pbc6_				0.12	0.07
*Number*	302		333	325	
χ^2^	49.70		23.04	0.17	
*df*	28		6	2	
*P*-value	0.007		0.001	0.9	
RMSEA	0.05		0.09	<0.0001	
SRMR	0.03		0.02	<0.01	
CFI	0.98		0.98	1.00	

Affective attitude (*AA*), instrumental attitude (*AI*), perceived norms (*PN*), and perceived behavioral control (*PBC*) are constructs in the reasoned action approach, an integrative framework for behavior analysis. This table describes 4 MIMIC models for causes, or γ (*att*_*1*_, *att*_*3*_, and *att*_*8*_ for *AA*; *att*_*4*_, *att*_*6*_, and *att*_*7*_ for *AI*; *in*_*1*_, *in*_*3*_, and *in*_*4*_ for *PN*; and *pbc*_*1*_, *pbc*_*2*_, *pbc*_*6*_ for *PBC*) related to each individual construct. Values for causes were generated using data from a survey querying current practices and future intentions for prudent use of antimicrobials on NY dairy farms. Each model describes the impact of causal indicators using the regression coefficients (γ). χ^2^, Chi-square; *df*, degrees of freedom, RMSEA, root mean square error of approximation; SRMR, standardized root mean square residual; CFI, Bentler’s comparative fit index.

## Discussion

Social construct modeling and analysis is now more commonly used to debrief and describe intentions to perform behaviors within the agricultural sector. In the structural equation models presented in this study, we were able to use the reasoned action approach to capture 53% of variance in intention to prudently use antimicrobials on dairies. Attitude, social pressures, and perceived behavioral control all were important determinants of intention, with perceived behavioral control as the primary determinant.

Overall attitude toward reduction in and prudent use of antimicrobials captured by surveys performed on dairies in the past 10 years has been positive [8, 24, 27, 37]. Within one of these publications [8], authors found that attitude explained 19% of the variance in applying selective treatment to cows at drying off, a recently enforced prudent use strategy on dairies in the Netherlands. In the current study we found that 29% of the variance was explained by two different attitudinal attributes: instrumental (economic) and affective (experiential). Instrumental attitude had the higher β estimate of the two, indicating that beneficial or rewarding outcomes, likely related to economics, positively motivate farmers’ intentions to adopt prudent use practices more so than affective attitude, or emotions that accompany the performance of the behavior, or attitudes based on moral thoughts.

The two highest scoring instrumental attributes, ‘health of the herd’ and ‘increased profitability’ are related, and are straight-forward in their economic influences on dairy farms. These considerations were also two of the four significant factors related to farmers’ mindsets in the survey on adoption of selective dry cow therapy [8]. The third highest scoring attribute in the current study, ‘decreased risk of residues’, could also stem from attitudes regarding economic outcomes. The Pasteurized Milk Ordinance guidelines outline a zero tolerance for concentrations of antibiotics in milk products [46]. Any positive bulk tank is penalized with permit suspension, responsibility for the value of milk in the entire load, and additional fines. In this manner, interventional programs should describe examples of successful prudent use strategies and the resulting increases in profitability and improvements in herd health, and additionally provide instruction on how such strategies are effective at residue avoidance.

Affective attitude indicators in the MIMIC model were ‘job satisfaction’, ‘increased milk production’, and ‘decreased risk of resistance’. The most influential underlying attitudinal belief was job satisfaction. This agrees with the research on selective dry-off treatment in the Netherlands, which showed that 87% of farmers considered themselves good farmers when they used less antimicrobials [8]. However, the opposite could occur when farmers view antimicrobial treatment as needed to improve animal welfare, regardless of how drugs are used. This was seen in survey respondents in Germany and the Netherlands who perceived extended treatment for mastitis as being a “good farmer” in the face of continued clinical signs and without concern of antibiotic residues or resistance [47]. In regards to the impact of increased milk production and decreased risk of resistance on attitudes of New York farmers, perhaps respondents were aware of, or had experienced improved herd health and decreased resistance as outcomes of prudent use practices. A majority (> 50%) of participating farms used Dairy Herd Information Association testing or had monthly or more frequent veterinary visits. This is suggestive of active herd health monitoring on these dairies, and perhaps frequent discussions of resistance with veterinarians. Attitudes regarding contributions to resistance may be location-dependent, however, as surveys performed in the southeastern US indicated that most farmers (86%) were not concerned that overuse of antibiotics could result in antibiotic resistance among workers, yet 40% of respondents to the same survey reported that they were very familiar with antibiotic resistance and 70% agreed that antibiotics become less effective with the frequency of use [48].

Our analysis indicates that highlighting any contributions of prudent use practices to job satisfaction, decreased risk of resistance, and subsequent increases in milk production can promote positive attitudes toward adopting these strategies. Certainly education on resistance including definitions and basic mechanisms would be beneficial. Job satisfaction likely encompasses but is not limited to improvements in animal welfare that may be associated with decreased need of antimicrobial administration, via continued efficacy or due to the practice of selective treatment.

In addition to attitude, social influences were a driver of dairy farmers’ intention to use antimicrobials prudently. A behavior model in the UK identified social influences as being the strongest drivers of intent to reduce antibiotic use [24]. The important injunctive norm attributes in the current study were ‘family members/friends’, ‘milk plant’, and ‘veterinarian.’ These findings agree with Kayitsinga et al. (2017), where 85% of farmers in the survey indicated that it is important or very important to consult with veterinarians for mastitis information and 55% said it is important or very important to get mastitis information from milk cooperatives [37]. Additionally, it is not uncommon to find veterinarians as one of the most influential referents for many intentions or behaviors on multiple farm types and locations. For example, in South Carolina 100% of farms indicated veterinarians were the preferred information source about antibiotics [48] and in the Netherlands, 71% of farmers saw their veterinary practitioner as the main advisor that encouraged them to reduce antimicrobial use on their farms [8]. The survey performed in the UK exploring knowledge, attitude, and practices on dairies indicated that the frequency of veterinary contact was associated with more ‘responsible’ treatment choices and increased knowledge of the repercussions of antibiotic use (*e*.*g*. resistance) [40]. Milk buyer (processor) is another common important referent in the current study as well as in Sok et al. (2015). While producers may indicate that certain groups or experts approve of reduction of antibiotic use, motivation to comply with these groups may be the reverse. This is likely one reason why milk consumers and/or government regulators were not important in our MIMIC models. Likewise, social pressures from veterinarians and other farmers can influence antimicrobial use in the opposite manner. Farmers were found to be sensitive toward social norms of other farmers and of veterinarians when recognizing that “thorough” treatment of mastitis cases involved treating for a duration longer than clinical signs; use of extended mastitis therapy was perceived as “being a good farmer” by other farmers and veterinarians [47]. This suggests that though mean values are high and positive within studies, antimicrobial use can be promoted (prudent use hindered) or discouraged by normative pressures. The wording within our survey did specify a direction for each referent’s influence, toward approval (positive) or disapproval (negative) of use of prudent practices. Our findings indicate that the approach for creating change in antimicrobial use is by fostering relationships and communication between farmers and those who approve of these practices, namely veterinarians, peers, and milk processors.

Finally, the primary driver for intention to use antimicrobials prudently in the current study was perceived behavioral control. In our MIMIC models, attributes focused on saving money on labor and treatment, ability to fit into the daily routine, and effectiveness with veterinary guidance. The importance of ‘saving money’ identifies that financial barriers determine a feeling of self-efficacy; limiting these barriers facilitates intention to use antibiotics prudently. Results also indicate that minimizing the time necessary for implementation of protocols would be beneficial. This suggests their need to be straightforward and easy to perform. Finally, communication in regards to knowledge and advice can be an extrinsic barrier to creating change, particularly between veterinarians and producers. Veterinary guidance was likely lowest of the three determinants due to many respondents already having active veterinary involvement (>50% had routine visits) and a majority having protocols reviewed often; respondents did not necessarily need future guidance to facilitate prudent use practices. Guidance is certainly important: individuals who receive more training prior to performing a behavior have higher success rates, and having a better understanding of illness and procedures contributes to higher self-efficacy which is positively related to better compliance [49–51].

One limitation of the current study is the low response rate (9.5%). Despite the low response rate, the distribution of responses reflects the current demographics and management style of farms in NY. Particularly, for farm size, the majority of farms have less than 100 cows. The larger farms, amounting to 6% of the respondents, however, were underrepresented. The 2017 census indicated that approximately 17% of NY dairy farms had greater than 500 milking cows [52]. This could be an important issue as larger farms were found to have statistically higher mean scores for attitudes toward reductions in antibiotic use than smaller farms when analysis was stratified by farm size in a similar study [37], This would indicate that the values in the current study may be underestimated. Differences in social pressures can be evident by farm size as well. Shortall et al. (2018) found that large-scale farmers associated “being a good farmer” and being “business minded” with detachment from cows and more focus on biosecurity, while smaller scale farmers expressed stronger emotional attachment and emphasized being a good neighbor [53]. Therefore, importance of social pressures and/or attitudes (*e*.*g*. job satisfaction) on intentions may deviate by farm size. Analysis was not completed by farm size in the current study; our results may be more reflective of owners of small dairy facilities. Differences may also exist in other demographics such as age, management style, etc. Given the small sample size and the decreased generalizability of a resulting interpretation, analysis was not explored by demographic groups.

Although anonymity was thoroughly disclosed in the cover sheet, due to the nature of the statements within the survey, there might have been potential for more recipients in favor of prudent use practices to submit responses. This could be reflected in lower blanket-treatment rates for clinical mastitis (30%) and dry cows (69%) than were calculated in the USDA-NAHMS survey (87% and 90%, respectively [19]). However, not all respondents treated all clinical cases and the majority of respondents treated all dry cows. Therefore, the current survey has captured responses of producers that may not practice prudent use in these particular areas. In the same vein: 1) socially desirable answers could have been reported by farmers, as antimicrobial use is a sensitive topic, leading to a bias in results and 2) presenting initial definitions of both “resistance” and “prudent use” could create anchoring bias. However, definitions, which were consistent with published information [9, 54], were offered to decrease misinterpretation for better accuracy [55] and to limit any stigma related to these terms in regards to the use of antibiotics in agriculture. A free-response format was used for our questionnaire; when this procedure is followed, it minimizes these biases [56].

Perhaps intent to use antimicrobials responsibly is captured well by the model because it is broadly accepted as an aim across many sectors of agriculture and veterinary medicine, including dairy. This is the case even though there is acknowledged uncertainty with respect to animal and public health risks such as resistance [6] and although there is awareness, this does not inevitably lead to behavioral change. For example, while 70% of dairy respondents in the UK survey agreed that ‘reducing use of antibiotics over the next year would be a good thing to do’, only 59% said they had the skills and knowledge needed to reduce antibiotics on their own farms [24]. This reflects one drawback to using an inherently restrictive social framework such as the reasoned action approach: an assumption that people with intentions will act without limitation (without the constraints such of time, demographic characteristics, unconscious habits, or irrational thoughts that may influence the freedom to act). Other arguments against the use of these models are: risks of confounding between each of the constructs as the definitions of each are not strict, and that perceived behavioral control predicts actual behavioral control [57]. Despite limitations, strong support for the modeling used comes from evidence describing the effectiveness of theory-based, applied interventions [56]. Though there may be correlation between variables, we tested for convergent and discriminant validity among them to decrease any potential confounding. We were able to show a predictive role for individual constructs and were able to capture a large proportion of the variability in intent to use prudently, indicating strength in the use of the theory in our approach. It may be possible to add more variables to the model to improve prediction of intentions, however, there is no acceptable or established level of explained variance [56]; measurement reliability, good fit, and high predictive validity was shown in the current analysis. The preceding models point out ways that we can approach change and develop programs by targeting the indicators that contribute to attitude, educating and motivating the referents that produce social pressures, and providing knowledge and tools that promote confidence and feelings of self-autonomy.

## Conclusion

Taken together, the findings in the current study indicate that attempts at promoting prudent change in antimicrobial use will need to work by approaching farmer-vet, farmer-processor, and farmer-peer relationships, aiming to build and maintain trust. These contacts should also serve as credible communicators, particularly instilling confidence and guidance in performing tasks necessary to reduce antimicrobial use, because changes in self-control rely on engagement over performance [58]. Recommendations may include education but also an ability of the veterinarian, peer, or processor to empower the farmer or enable him or her to develop his or her own competencies for prudent use by encouraging co-creation of knowledge and reflection by different stakeholders on the dairy, allowing the farmer to take responsibility for his or her current situation. Major barriers to seeking out antimicrobial use protocols are lack of time and limited finances, particularly for implementation and veterinarian consultation, which can be problematic given the findings in the current study. However, increasingly, animal health companies and veterinary agencies are offering fully funded seminars for producers on prudent antimicrobial use, sharing producer anecdotal successes and positive outcomes from clinical research trials. To promote positive attitudes, these successes and trials should reference 1) any economic advantages by presenting data on increased profitability, 2) decreases in resistance and residue risks, 3) improvements in herd health or assurance of low risks to animal welfare, and 4) contributions to job satisfaction.

## Supporting information

S1 Appendix Survey querying current practices and future intentions for prudent use of antimicrobials on NY dairy farms developed using guidelines for the reasoned action approach. (PDF)

S1 Table Farm, management, and demographic details of responders to a survey on prudent antimicrobial use practices and intentions on New York dairy farms (n = 411). ^1^Somatic cell count is the concentration of somatic cells in milk, including white blood cells, that is often used as an indicator of milk quality.
(DOCX)

S2 Table Respecification statistics for structural equation model. Attitude, perceived norms, and perceived behavioral control are constructs in the reasoned action approach, an integrative framework for behavior analysis. This table describes the respecification of structural equation models. Values for direct measures were generated using data from a survey querying current practices and future intentions for prudent use of antimicrobials on NY dairy farms. The saturated model included all questions corresponding to each construct, while the final model discarded questions outlined in the manuscript as well as identified two separate constructs for attitude. Abbreviations of indices with their acceptable cutoffs χ^2^, Chi-square; RMSEA, root mean square error of approximation (≤ 0.06); CFI, Bentler’s comparative fit index (≥ 0.95); SRMR, standardized root mean square residual (≤ 0.08); CR, composite reliability (> 0.95); AVE, average variance extracted (≥ 0.5).
(DOCX)
