# Supplementary material for: Understanding the effect of producers’ attitudes, perceived norms, and perceived behavioral control on intentions to use antimicrobials prudently on New York dairy farms
Source: PLoS One. 2019 Sep 11;14(9):e0222442. doi: 10.1371/journal.pone.0222442 (PMC6738616; doi:10.1371/journal.pone.0222442)
Supplement: S1 Appendix — (PDF) [file pone.0222442.s001.pdf]

Cornell University
College of Veterinary Medicine

Dear Participant,

Thank you in advance for taking the time to contribute to a project very important to the dairy industry and current research knowledge. We are veterinarians in the Population Medicine department at the Cornell Veterinary School and we are reaching out to NY dairy producers to ask for answers to a brief survey. Compensation will be provided to many participants.

More detail is found below about purpose, confidentiality, and compensation. We highly encourage the person with the most knowledge about decisions regarding antibiotic use on your dairy to fill out this questionnaire. This survey can be taken on-line or on paper. Again, your participation is greatly appreciated.

Informed Consent

Who are we: Cornell University College of Veterinary Medicine working closely with Quality Milk Production Services (QMPS).

We are asking you to participate in a research survey. We anticipate it should take you approximately 10-15 minutes of your time to complete.

Project title: "Survey of NY dairies: factors that influence antibiotic use"

What the study is about

The purpose of this research is to understand what factors influence treatment decisions when using antibiotics on dairies.

What we will ask you to do

If you choose to participate, you will be asked to answer questions about antibiotic use decision-making on your dairy, your thoughts on current regulations, along with collection of demographic information.

Benefits

The answers retrieved will help guide veterinarians and consultants to establish effective ways of implementing antibiotic protocols on dairies.

Compensation for participation

We would like to compensate you for your time filling out this questionnaire. Every 10th participant will receive a $20 amazon gift card. You will be asked to provide your email address on a separate page so upon receipt the survey responses and email address will be separate and cannot be linked.

Privacy/Confidentiality/Data Security

The responses that you provide in this study will be kept completely confidential and anonymous (i.e., your name will not be associated with your responses in any way). We will not publish individual responses.

Taking part is voluntary
Your involvement in this study is voluntary. You may refuse to participate before the study begins, discontinue at any time, or skip any questions that may make you feel uncomfortable, with no penalty.

If you have questions
The main researcher conducting this study is Dr. Daryl Nydam at Cornell University. If you have questions, you may contact Daryl Nydam at dvn2@cornell.edu. If you have any questions or concerns regarding your rights as a subject in this study, you may contact the Institutional Review Board (IRB) for Human Participants at 607-255-6182 or access their website at http://www.irb.cornell.edu. You may also report your concerns or complaints anonymously through Ethicspoint online at www.hotline.cornell.edu or by calling toll free at 1-866-293-3077.

You may complete the attached survey on paper, or online by scanning the QR code or typing in the short URL below:

https://goo.gl/VdR6NV

Statement of Consent
I have read the above information, and have received answers to any questions I asked. I consent to take part in the study.

By entering ***your initials*** *in the box below, you are effectively providing your signature, indicating that all the information on this form is true and accurate, to the best of your knowledge.*

Date (MM/DD/YYYY)

Demographics

1. Job title of person filling in questionnaire?

- ○ Owner
- ○ Manager
- ○ Non-management farm employee
- ○ Herd veterinarian
- ○ Other ______

2. What is your current age?

______

3. What is your highest level of education?

- ○ Pre-High School
- ○ High School/GED
- ○ Some College
- ○ Bachelors Degree
- ○ Graduate/Professional School
- ○ Prefer not to answer

We would like to know a little more about the farm you represent

4. Pick one. My farm is:

- ○ Organic
- ○ Conventional

5. How many years has this business been in operation?

______

6. How many lactating cows on all of your facilities?

______

7. What is the average fat corrected milk per cow per day across the herd?

- ○ <70 lbs
- ○ 70-80 lbs
- ○ 81-90 lbs
- ○ >90 lbs

8. What is your most recent bulk tank SCC (cells/ml)?
Example: 100,000

9. Do you participate in DHIA: Dairy Herd Improvement Association, testing?

- Yes
- No

10. What is the predominant type of housing for lactating cows on your dairy

- Freestall
- Tie Stall
- Pasture
- Bedded pack
- Other

11. The last time written treatment protocols were reviewed by a veterinarian was:

- In the last 3 months
- In the last 6 months
- In the last year
- In over a year
- Our dairy does not have written protocols

12. Do you treat all cows with clinical mastitis with antibiotics?

- Yes
- No

13. Do you treat all cows with antibiotics at dry off (dry tubes, for example)?

- Yes
- No

14. If you have routine/schedule times when a veterinarian visits how often are they?

- One time or more per week
- Twice per month
- Every other month
- Less than 6 times per year
- We do not have regular routine visits, only for sick cows/emergencies

Questionnaire

Loss of effectiveness of an antibiotic is one of the effects of antibiotic resistance. To preserve the effectiveness of an antibiotic by using it only to treat the affected animals that will benefit from the treatment, is also called "prudent use of antibiotics". This can be achieved by using diagnostics, using cattle records to determine which cows to treat and not treating suspect viral infections. Overall this might lead to a reduction of the use of antibiotics.

<u>The following questions refer to decisions made on your farm over the next two years.</u>

15. Please indicate how likely:
Using antibiotics prudently will:

	Not at all likely<br>1	2	>><br>3	>><br>4	Very likely<br>5
Increase milk production;	○	○	○	○	○
Be cost-effective;	○	○	○	○	○
Add to your job satisfaction;	○	○	○	○	○
Increase the health of your herd;	○	○	○	○	○
Enhance your reputation in the dairy industry;	○	○	○	○	○
Increase the profitability of your farm;	○	○	○	○	○
Decrease the risk of residues in the bulk tank;	○	○	○	○	○
Decrease the risk of antibiotic resistance on your farm.	○	○	○	○	○

16. How important are the following motives for using antibiotics prudently:

	Not at all important<br>-2	-1	<<<br>0	>><br>1	Extremely important<br>2
Increased milk production of your cows;	○	○	○	○	○
Cost-effectiveness;	○	○	○	○	○
Your job satisfaction;	○	○	○	○	○
The health of your herd;	○	○	○	○	○
Your reputation in the dairy industry;	○	○	○	○	○
The profitability of your farm;	○	○	○	○	○
Decreased risk of residues in the bulk tank;	○	○	○	○	○
Decreased risk of antibiotic resistance on your farm.	○	○	○	○	○

The following questions refer to decisions made on your farm over the next two years.

17. Please indicate on the scale below whether using antibiotics prudently would be:

Disadvantageous	○	○	○	○	○	Advantageous
Unsatisfying	○	○	○	○	○	Satisfying
Unnecessary	○	○	○	○	○	Necessary
Unimportant	○	○	○	○	○	Important
Unpleasant	○	○	○	○	○	Pleasant

Questions 18 and 19 are linked.

18. Do you think the following people would approve or disapprove of you using antibiotics prudently?

	Strongly disapprove -2	-1	<< 0	>> 1	Strongly approve 2	Don't know
Family members and/or friends;	○	○	○	○	○	○
Neighboring farmers;	○	○	○	○	○	○
Veterinarians;	○	○	○	○	○	○
Your milk plant;	○	○	○	○	○	○
Milk consumers;	○	○	○	○	○	○
Scientists/researchers;	○	○	○	○	○	○
Government regulators.	○	○	○	○	○	○

19. How important is their opinion to you in regards to your prudent use of antibiotics?

	Not at all important 1	2	>> 3	>> 4	Extremely important 5
Family members and/or friends;	○	○	○	○	○
Neighboring farmers;	○	○	○	○	○
Veterinarians;	○	○	○	○	○
Your milk buyer;	○	○	○	○	○
Milk consumers;	○	○	○	○	○
Scientists/researchers;	○	○	○	○	○
Government regulators.	○	○	○	○	○

20. Please indicate your level of agreement with the following statements:

	Strongly disagree -2	-1	<< 0	>> 1	Strongly agree 2
**Most people who have something to do with my farm** expect me to use antibioics prudently;	○	○	○	○	○
**The people in the dairy industry whose opinions I value** would approve of me using antibiotics prudently;	○	○	○	○	○
**Most people who are important to me** think that I should use antibiotics prudently.	○	○	○	○	○

The following questions refer to decisions made on your farm over the next two years.

21. Do you expect that the following people are going to use antibiotics prudently:

	Unlikely -2	-1	<< 0 >>	1	Extremely likely 2	Don't know
Leading dairy farms;	○	○	○	○	○	○
Your farmer friends/neighbors;	○	○	○	○	○	○
Farms serviced by my veterinarian;	○	○	○	○	○	○
Other livestock (non-dairy) farms;	○	○	○	○	○	○
Farms in other countries.	○	○	○	○	○	○

22. How important are the successful actions of the following people in your considerations to use antibiotics prudently:

	Not at all important 1	2	>> 3 >>	4	Extremely important 5
Leading dairy farms;	○	○	○	○	○
Your farmer friends/neighbors;	○	○	○	○	○
Farms serviced by my veterinarian;	○	○	○	○	○
Other livestock (non-dairy) farms;	○	○	○	○	○
Farms in other countries.	○	○	○	○	○

23. Please indicate how likely using antibiotics prudently will:

	Not very likely 1	2	>> 3 >>	4	Extremely likely 5
Fit into your daily work routine;	○	○	○	○	○
Save money on treatment and labor;	○	○	○	○	○
Be feasible as you know exactly what should be improved;	○	○	○	○	○
Would be easy to see changes in animal health in the short term;	○	○	○	○	○
Would be easy to see changes in revenue flow in the short term;	○	○	○	○	○
Only be effective with veterinary or consultant guidance;	○	○	○	○	○
Be compensated with premiums.	○	○	○	○	○

24. Will the following points make it easier or more difficult for you to use antibiotics prudently:

	Extremely difficult -2	-1	<< 0 >>	1	Extremely easy 2
Fitting into daily work routine;	○	○	○	○	○
Labor/money that is saved;	○	○	○	○	○
Knowing exactly what should be improved;	○	○	○	○	○
Achieving animal health benefits in the short term;	○	○	○	○	○
Achieving economic benefits in the short term;	○	○	○	○	○
Availability of veterinary or consultant guidance;	○	○	○	○	○
Compensation with premiums;	○	○	○	○	○
Having protocols computer automated.	○	○	○	○	○

<u>The following questions refer to decisions made on your farm over the next two years.</u>

25. Please indicate your level of agreement with the following statements:

	Strongly disagree -2	-1	<< >> 0	1	Strongly agree 2
**I have the possibility to** use antibiotics prudently;	○	○	○	○	○
**If I wanted to, I could** use antibiotics prudently;	○	○	○	○	○
**It is up to me whether I** use antibiotics prudently;	○	○	○	○	○
**I am confident that I can** use antibiotics prudently;	○	○	○	○	○
**I will try to** use antibiotics prudently;	○	○	○	○	○
**I intend to** use antibiotics prudently;	○	○	○	○	○
**I plan to** use antibiotics prudently.	○	○	○	○	○

26. When you think about being a dairy farmer and managing all aspects of your dairy farm, how important are the following:

	Very unimportant -2	-1	<< >> 0	1	Very Important 2
Getting as much profit from the herd as I can;	○	○	○	○	○
Having a satisfying job;	○	○	○	○	○
Improving the herd's physical performance;	○	○	○	○	○
Having the highest herd welfare standards;	○	○	○	○	○
Managing the farm more cost-effectively;	○	○	○	○	○
Being seen as a dairy farmer/employee with a good reputation.	○	○	○	○	○

27. Approximately how many milking cows are treated with antibiotics for mastitis per week?

28. If you have any additional comments we would like to read them. Please write below:

Thank you for your time and patience in filling out this questionnaire.

All data provided in this survey will be confidential. Your survey responses and email address will not be linked.

Please turn the page for your chance to win a $20 Amazon gift card!

Project title: “Survey of NY dairies: factors that influence antibiotic use”

Principal Investigator: Daryl Nydam,

Population Medicine and Diagnostic Sciences
Cornell University College of Veterinary Medicine
dvn2@cornell.edu

Compensation for participation

We would like to compensate you for your time filling out this questionnaire. Every 10th participant will receive a $20 amazon gift card. Please provide your email address so upon receipt the survey responses and email address will be separate and cannot be linked.

Your Name (First, Last)

Your Email Address
