# Supplementary material for: Understanding the effect of producers’ attitudes, perceived norms, and perceived behavioral control on intentions to use antimicrobials prudently on New York dairy farms
Source: PLoS One. 2019 Sep 11;14(9):e0222442. doi: 10.1371/journal.pone.0222442 (PMC6738616; doi:10.1371/journal.pone.0222442)
Supplement: S1 Table — 1Somatic cell count is the concentration of somatic cells in milk, including white blood cells, that is often used as an indicator of milk quality. (DOCX) [file pone.0222442.s002.docx]

| **Question/Answer** | **Number (%)** | **Question/Answer** | **Number (%)** |
| --- | --- | --- | --- |
| **Job title of respondent** | | **Participation in Dairy Herd Information Association testing** | |
| Owner | 373 (90.8) | Yes | 282 (68.6) |
| Manager | 28 (6.8) | No | 126 (30.7) |
| Other (employee or no answer) | 10 (2.4) | No answer | 3 (0.7) |
| **Current age of respondent (years)** | | **Treat all cows with clinical mastitis with antibiotics** | |
| < 40 | 89 (21.7) | No | 285 (69.3) |
| 40 to 60 | 193 (47.0) | Yes | 122 (29.7) |
| > 60 | 113 (27.5) | No answer | 4 (1.0) |
| Prefer not to answer | 16 (3.9) |  |  |
| **Highest level of education of respondent** | | **Frequency of veterinary visits** | |
| Pre-High School  High school | 45 (11.0)  99 (24.1) | No regular routine visits, only for sick cows/emergencies | 114 (27.7) |
| Some College | 117 (28.5) | Twice per month, or more | 94 (22.9) |
| Bachelor’s Degree | 120 (29.2) | Once per month | 69 (16.8) |
| Graduate/Professional School | 17 (4.1) | One time or more per week | 69 (16.8) |
| Prefer not to answer/no answer | 13 (3.2) | Every other month/ less frequently, but routine | 63 (15.3) |
|  |  | No answer | 2 (0.5) |
| **Farm type** | | **Treat all cows with antibiotics at dry off** | |
| Conventional | 364 (88.6) | Yes | 283 (68.9) |
| Organic | 47 (11.4) | No | 126 (30.7) |
|  |  | No answer | 2 (0.5) |
| **Number of years dairy has been in operation** | | **Predominant type of housing for lactating cows on dairy** | |
| 0-30 | 148 (36.0) | Freestall | 191 (46.5) |
| 31-60 | 157 (38.2) | Tie Stall | 168 (40.9) |
| 61-90 | 49 (11.9) | Pasture | 30 (7.3) |
| 91-120 | 39 (9.5) | Other | 20 (4.9) |
| > 120 | 16 (3.9) | No answer | 2 (0.5) |
| No answer | 2 (0.5) |  |  |
| **Number of lactating cows** |  | **Last time written treatment protocols were reviewed by a veterinarian** | |
| < 50 | 79 (19.2) | Our dairy does not have written protocols | 116 (28.2) |
| 50-99 | 155 (37.7) | In the last 6 months | 146 (35.5) |
| 100-499 | 105 (25.5) | In the last year | 93 (22.6) |
| > 499 | 68 (16.6) | In over a year | 50 (12.2) |
| No answer | 4 (1.0) | No answer | 6 (1.5) |
| **Average fat-corrected milk production per cow per day across the herd (lbs)** | | **Most recent bulk tank somatic cell count (cells/ml)^1^** | |
| < 70 | 159 (38.7) | < 100,000 | 95 (23.1) |
| 70 to 80 | 104 (25.3) | 100,000-200,000 | 220 (53.5) |
| 80 to 90 | 86 (20.9) | 201,000- 300,000 | 66 (16.1) |
| > 90 | 48 (11.7) | > 300,000 | 16 (3.9) |
| No answer | 14 (3.4) | No answer | 14 (3.4) |
