# Supplementary material for: Understanding the effect of producers’ attitudes, perceived norms, and perceived behavioral control on intentions to use antimicrobials prudently on New York dairy farms
Source: PLoS One. 2019 Sep 11;14(9):e0222442. doi: 10.1371/journal.pone.0222442 (PMC6738616; doi:10.1371/journal.pone.0222442)
Supplement: S2 Table — Attitude, perceived norms, and perceived behavioral control are constructs in the reasoned action approach, an integrative framework for behavior analysis. This table describes the respecification of structural equation models. Values for direct measures were generated using data from a survey querying current practices and future intentions for prudent use of antimicrobials on NY dairy farms. The saturated model included all questions corresponding to each construct, while the final model discarded questions outlined in the manuscript as well as identified two separate constructs for attitude. Abbreviations of indices with their acceptable cutoffs χ2, Chi-square; RMSEA, root mean square error of approximation (≤ 0.06); CFI, Bentler’s comparative fit index (≥ 0.95); SRMR, standardized root mean square residual (≤ 0.08); CR, composite reliability (> 0.95); AVE, average variance extracted (≥ 0.5). (DOCX) [file pone.0222442.s003.docx]

|  | Statistic | | | | | |
| --- | --- | --- | --- | --- | --- | --- |
| **Model or Construct** | **χ^2^** | **RMSEA** | **CFI** | **SRMR** | **CR** | **AVE** |
| Saturated model | 360.53 | 0.10 | 0.92 | 0.05 | n/a | n/a |
| Attitude, as one construct | n/a | n/a | n/a | n/a | 0.87 | 0.58 |
| Instrumental attitude | n/a | n/a | n/a | n/a | 0.74 | 0.89 |
| Affective attitude | n/a | n/a | n/a | n/a | 0.64 | 0.64 |
| Perceived behavioral control | n/a | n/a | n/a | n/a | 0.82 | 0.54 |
| Perceived behavioral control, with removals | n/a | n/a | n/a | n/a | 0.61 | 0.45 |
| Intention | n/a | n/a | n/a | n/a | 0.93 | 0.81 |
| Intention, with removals | n/a | n/a | n/a | n/a | 0.92 | 0.86 |
| Final model | 77.95 | 0.05 | 0.99 | 0.03 | n/a | n/a |
